# Inhibition of YAP1 activity ameliorates acute lung injury through promotion of M2 macrophage polarization

**DOI:** 10.1002/mco2.293

**Published:** 2023-06-05

**Authors:** Lu Liang, Wenyan Xu, Ao Shen, Xiaomei Fu, Huiyu Cen, Siran Wang, Zhongxiao Lin, Lingmin Zhang, Fangyu Lin, Xin Zhang, Na Zhou, Jishuo Chang, Zhe‐Sheng Chen, Chuwen Li, Xiyong Yu

**Affiliations:** ^1^ Guangzhou Municipal and Guangdong Provincial Key Laboratory of Molecular Target & Clinical Pharmacology The State & NMPA Key Laboratory of Respiratory Disease School of Pharmaceutical Sciences & The Fifth Affiliated Hospital Guangzhou Medical University Guangzhou China; ^2^ Department of Preventive Dentistry Affiliated Stomatology Hospital of Guangzhou Medical University Guangdong Engineering Research Center of Oral Restoration and Reconstruction Guangzhou Key Laboratory of Basic and Applied Research of Oral Regenerative Medicine Guangzhou China; ^3^ State Key Laboratory of Quality Research in Chinese Medicine Macau University of Science and Technology Avenida Wailong Taipa Macau China; ^4^ Department of Ophthalmology B5500 Clinic B 1365B Clifton Road NE Emory University Atlanta Georgia USA; ^5^ Department of Pharmaceutical Sciences Institute for Biotechnology College of Pharmacy and Health Sciences St. John's University Queens New York USA

**Keywords:** Yes‐associated protein 1, macrophage polarization, acute lung injury, pulmonary inflammation

## Abstract

The balance of M1/M2 macrophage polarization plays an important role in regulating inflammation during acute lung injury (ALI). Yes‐associated protein (YAP1) is a key protein in the Hippo–YAP1 signaling pathway and is involved in macrophage polarization. We aimed to determine the role of YAP1 in pulmonary inflammation following ALI and regulation of M1/M2 polarization. Pulmonary inflammation and injury with upregulation of YAP1 were observed in lipopolysaccharide (LPS)‐induced ALI. The YAP1 inhibitor, verteporfin, attenuated pulmonary inflammation and improved lung function in ALI mice. Moreover, verteporfin promoted M2 polarization and inhibited M1 polarization in the lung tissues of ALI mice and LPS‐treated bone marrow‐derived macrophages (BMMs). Additionally, siRNA knockdown confirmed that silencing *Yap1* decreased chemokine ligand 2 (CCL2) expression and promoted M2 polarization, whereas silencing large tumor suppressor 1 (*Lats1*) increased CCL2 expression and induced M1 polarization in LPS‐treated BMMs. To investigate the role of inflammatory macrophages in ALI mice, we performed single‐cell RNA sequencing of macrophages isolated from the lungs. Thus, verteporfin could activate the immune‐inflammatory response, promote the potential of M2 macrophages, and alleviate LPS‐induced ALI. Our results reveal a novel mechanism where YAP1‐mediated M2 polarization alleviates ALI. Therefore, inhibition of YAP1 may be a target for the treatment of ALI.

## Graphical abstract

Proposed mechanism by which YAP1 inhibition promotes M2 polarization in ALI. LPS administration induces pulmonary inflammation and upregulates YAP1 expression in ALI mice. The inhibition of YAP1 activity by verteporfin attenuates lung injury and improves pulmonary function, which is associated with macrophage polarization. YAP1 deficiency decreases CCL2 levels and promotes macrophage polarization towards the M2 phenotype. Graphical abstract was created with BioRender.com under an academic laboratory subscription.

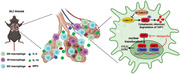

## 1 INTRODUCTION

Acute lung injury (ALI), also known as mild acute respiratory distress syndrome (ARDS) according to the Berlin Definition, represents a widespread type of pulmonary dysfunction due to a variety of pulmonary and generalized acute diseases, such as pneumonia, sepsis, trauma, and gastric aspiration.^1^, ^2^ Despite progress in the understanding of this disease, ALI‐related mortality remains high.^3^, ^4^ ALI is characterized by severe hypoxemia and noncardiogenic pulmonary edema secondary to uncontrolled inflammation.^2^ Therefore, accelerating the resolution of lung inflammation and promoting lung repair are promising strategies for ALI treatment.^3^, ^4^ To effectively cure ALI, understanding the mechanisms and signaling pathways involved in this process is necessary.

Numerous effector and target cells, particularly for lung macrophages, are associated with the pathophysiology mechanism of ALI/ARDS.^5^ Lung macrophages function as the first line of the innate immune system and defend against microbes and airborne particles.^6^, ^7^ Previous studies have reported that macrophages participate in the pathogenesis of ALI/ARDS, including the regulation of pulmonary inflammation and the repair of damaged lung tissues.^6^, ^8^, ^9^ Macrophages can be polarized into classically activated (M1) or alternatively activated (M2) macrophages in different microenvironments.^10^, ^11^, ^12^ In the acute exudative phase of ALI/ARDS, lung macrophages are polarized into M1 macrophages. Continuous M1 polarization can produce reactive oxygen species (ROS), nitric oxide (NO), interleukin 1 (IL‐1), and tumor necrosis factor α (TNF‐α) to promote the inflammatory response.^13^ In contrast, M2 macrophages are key factors in the regulation of lung damage and tissue repair in the rehabilitation phase of ALI/ARDS.^14^ Polarization of M1/M2 macrophages is a tightly controlled process, and an imbalance in M1/M2 polarization may induce excessive production of proinflammatory cytokines and lead to aggravated inflammation.^10^, ^11^, ^12^ Thus, controlling the polarization of macrophages may contribute to the prognosis of ALI.

The Hippo–YAP1 pathway is an evolutionarily conserved signaling pathway that controls a series of biological processes, such as tissue growth and organ size. Critical regulators of the Hippo–YAP1 signaling pathway include large tumor suppressor kinases 1/2 (LATS1/2), mammalian Ste20‐like kinases 1/2 (Mst1/2), and downstream components, including transcriptional coactivator with PDZ‑binding motif (TAZ) and Yes‐associated protein 1 (YAP1), which interact with TEA/ATTS domain transcription factors (TEADs).^15^, ^16^, ^17^, ^18^ YAP1 activity is mainly controlled by subcellular translocation, which is a consequence of phosphorylation.^15^, ^16^, ^17^, ^18^ When the Hippo signaling pathway is activated, phosphorylated Mst1/2 activates LATS1/2, which in turn promotes YAP1 phosphorylation, leading to inhibition of YAP1 activity via cytoplasmic retention. Conversely, the kinase activities of Mst1/2 and LATS1/2 are suppressed when the Hippo signaling pathway is turned off, leading to YAP1 dephosphorylation and translocation into the nucleus where it interacts with TEADs to promote the expression of target genes.^15^, ^16^, ^17^, ^18^ Increasing evidence has demonstrated that YAP1 is involved in macrophage‐mediated inflammatory responses.^19^, ^20^, ^21^ Inhibition of YAP1 activity suppresses inflammation by preventing lipopolysaccharide (LPS)‐stimulated nuclear factor kappa B (NF‐κB) nuclear translocation and activation in macrophages.^21^ In contrast, YAP1 activation aggravates inflammatory bowel disease by regulating the polarization of M1/M2 macrophages.^22^ Moreover, previous studies have indicated that YAP1 activation is closely associated with the pathogenesis of macrophage‐mediated inflammation.^19^, ^20^, ^21^, ^22^

Currently, it is controversial whether YAP1 participated in ALI‐induced imbalance of M1/M2 polarization and inflammation‐associated processes. The aim of this study was to investigate the role of YAP1 in ALI. Overall, these results demonstrate that YAP1 in lung macrophages is involved in the imbalance of M1/M2 polarization and pulmonary inflammation during the development of ALI. Verteporfin (VER) has been identified as a YAP/TEAD inhibitor^23^ and has been used to evaluate the role of YAP1 in ALI. Our current investigations revealed that inhibiting YAP1 expression using VER can effectively combat inflammation, attenuate lung injury, and improve pulmonary function associated with macrophage polarization. These results suggest that VER has the potential to function as a therapeutic agent against inflammatory ailments.

## 2 RESULTS

### 2.1 LPS‐induced ALI is associated with increased YAP1 activity in mice

**FIGURE 1:**
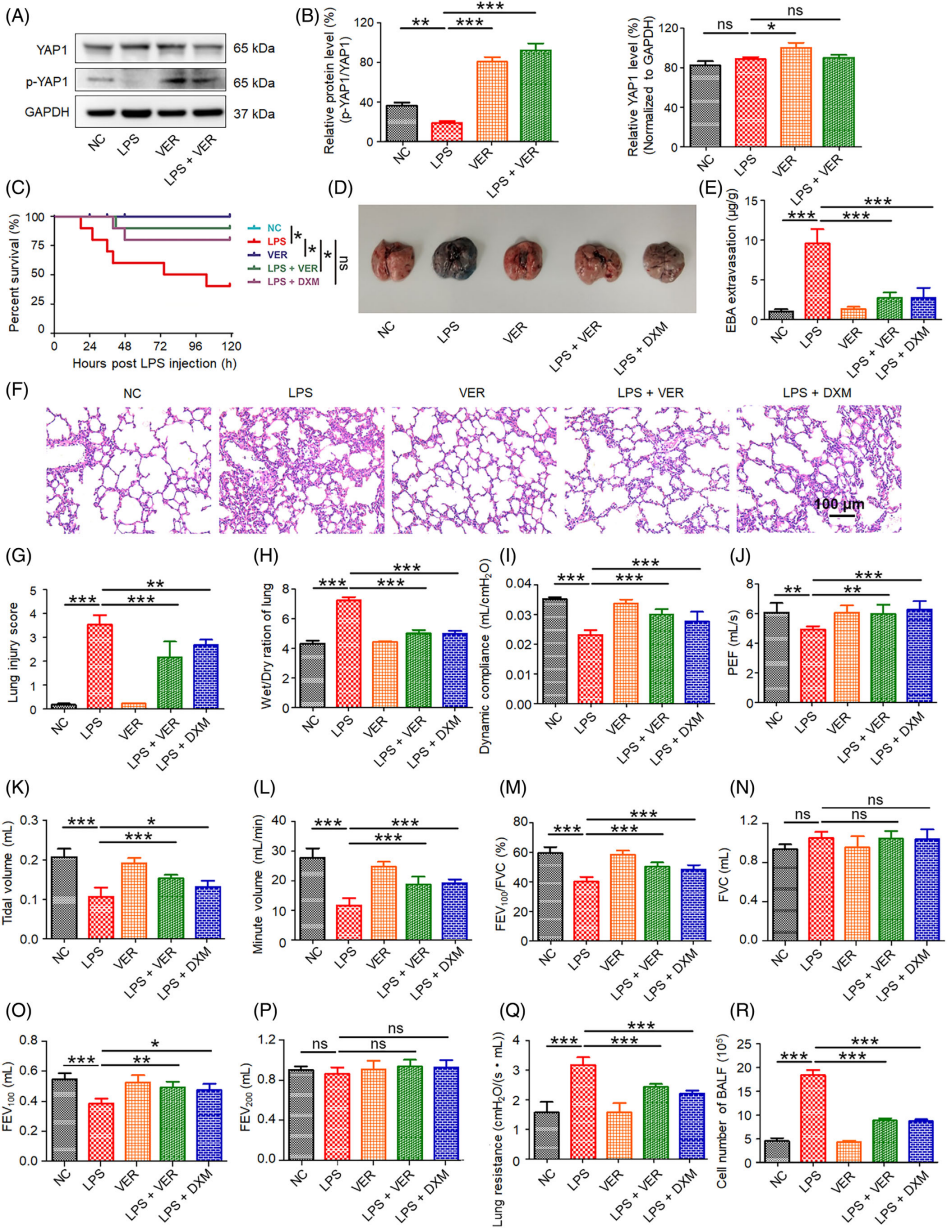
Inhibition of YAP1 activity improves survival rate and attenuates lung injury in ALI model mice. The pulmonary function was investigated, lung tissues and BALF were collected at 72 h after LPS administration. Western blotting (A) and densitometric quantification (B) of p‐YAP1 and YAP1 (*n* = 5). (C) The survival rates were recorded and calculated for 120 h (*n* = 10). Representative images of lung samples in EBA extravasation assay (D) and quantification of EBA (E). Histological analysis (F), lung injury score (G), wet/dry ratio (H), dynamic compliance (I), PEF (J), tidal volume (K), minute volume (L), FEV_100_/FVC % (M), FVC (N), FEV_100_ (O), FEV_200_ (P), lung resistance (Q), and cell number of BALF (R) (*n* = 5). All data are presented as the mean ± SD. ns, no significance; *p < 0.05, **p < 0.01, ***p < 0.001 between two groups. EBA, Evans Blue albumin; PEF, peak expiratory flow; FEV_100_, forced expiratory volume expired during the first 100 ms of expiration; FVC, forced vital capacity; FEV_100_, forced expiratory volume expired during the first 100 ms of expiration; FEV_200_, forced expiratory volume expired during the first 200 ms of expiration; BALF, bronchoalveolar lavage fluid.

In the current study, we first determined whether alterations in YAP1 activity were associated with lung injury. YAP1 activity is controlled by phosphorylation which inhibits YAP1 nuclear translocation and transactivation activity. The p‐YAP1/YAP1 ratio was significantly decreased in the lung tissues of LPS‐induced ALI mice, suggesting increased YAP1 activity in the lungs (Figures 1A and B). These results indicated that LPS altered YAP1 activity in the Hippo–YAP1 signaling pathway in the lung tissues of ALI mice. To further test the function of YAP1 in ALI, we evaluated the effect of VER. VER, a United States Food and Drug Administration‐approved drug, is used as a photosensitizer in photodynamic therapy for the treatment of certain eye diseases. Additionally, it is a specific inhibitor of YAP1 that disrupts YAP–TEAD interactions.^23^, ^24^ In this study, we used VER as a YAP1 inhibitor. Treatment with VER increased the p‐YAP1/YAP1 ratio, indicating that YAP1 activity was inhibited (Figures 1A and B).

### 2.2 Inhibition of YAP1 activity improves survival rate, attenuates lung injury, and improves pulmonary function in ALI mice

We further investigated the influence of YAP1 on the survival rate and pulmonary function of ALI mice. VER (100 mg/kg, intraperitoneally administered) was used to evaluate ALI‐induced mortality in mice. As shown in Figure 1C, compared with the normal control group, the survival rate of mice in the ALI group was only 40% within 120 h. Conversely, VER significantly improved the survival rate, from 40 to 90%, 120 h after LPS challenge. When dexamethasone sodium phosphate (DXM), a widely prescribed anti‐inflammatory drug, was injected intraperitoneally into the mice, the survival of LPS‐challenged mice also improved (Figure 1C). However, VER exhibited greater improvement than DXM. These results indicated that VER treatment protected mice against ALI‐induced mortality.

We evaluated pulmonary function in the ALI model with or without VER treatment. First, lung capillary leakage was assessed by determining the Evans Blue albumin (EBA) extravasation in lung tissues. As shown in Figures 1D and E, ALI induced a significant increase in EBA extravasation. In contrast, VER and DXM treatment markedly suppressed EBA extravasation, indicating that VER treatment inhibited lung capillary leakage and preserved the lung capillary function in ALI mice. Compared with the uninfected control mice, alveolar space collapse and gradually aggravated histopathological injury were observed in the lungs of infected mice and there was a high degree of inflammatory cell infiltration in the lung tissues (Figure 1F). In ALI mice, treatment with VER and DXM significantly attenuated tissue injuries (Figure 1F), thus the lung injury scores were decreased (Figure 1G). Lung weight and dry ratio (W/D) was used for the evaluation of pulmonary edema. The ALI mice exhibited a higher W/D than those in the VER and DXM groups (Figure 1H). Dynamic compliance, peak expiratory flow (PEF), tidal volume, minute volume, forced expiratory volume in the first 100 ms of expiration/forced vital capacity (FEV_100_/FVC %) and FEV_100_ were significantly decreased in LPS‐induced mice compared with the control groups (*p* < 0.05). These decreases were reversed and the beneficial effects of VER and DXM on dynamic compliance, PEF, tidal volume and minute volume, FEV_100_/FVC and FEV_100_ were found (Figures 1I–P). Meanwhile, we observed the significant increases of lung resistance and the cell number of bronchoalveolar lavage fluid (BALF) in LPS‐induced mice. VER and DXM treatment decreased the lung resistance and the cell number of BALF (Figures 1Q and R). These data suggested that VER was able to attenuate the effects of LPS on pulmonary function.

### 2.3 Biodistribution analysis of VER with in vivo tracking system

The biodistribution of VER was observed using an in vivo tracking system. As shown in Figures S1A and S1B, VER was mainly distributed in the lung. A large amount of VER was captured in the lungs, indicating that it could effectively relieve lung inflammation and improve pulmonary function.

### 2.4 Inhibition of YAP1 activity ameliorates inflammation in ALI mice

**FIGURE 2:**
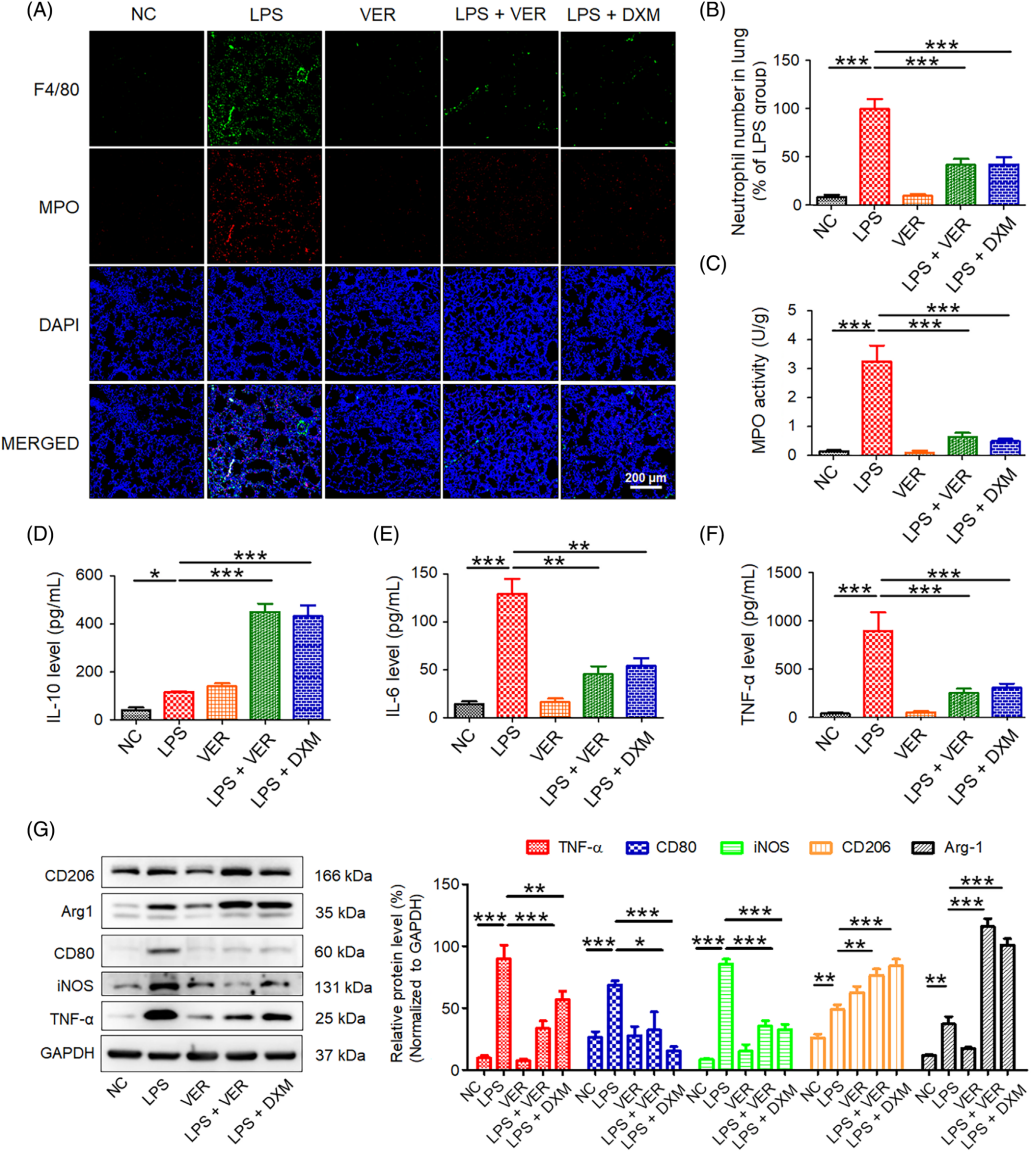
Inhibition of YAP1 activity ameliorates inflammation in ALI model mice. The lung tissues and BALF were collected at 72 h after LPS administration. (A) Immunofluorescence staining of the macrophage marker F4/80 (green) and the neutrophil marker MPO (red). Nuclei were counterstained with DAPI (blue). (B) Quantification of positive neutrophils. (C) Determination of MPO activity. The levels of IL‐10 (D), IL‐6 (E), and TNF‐α (F) were determined by ELISA assays. (G) Western blotting and densitometric quantification of TNF‐α, CD80, iNOS, CD206, and Arg1. All data are presented as the mean ± SD (*n* = 5). ns, no significance; *p < 0.05, **p < 0.01, ***p < 0.001 between two groups. MPO, myeloperoxidase; TNF‐α, tumor necrosis factor α; IL‐6, interleukin 6; IL‐10, interleukin 10. CD206, mannose receptor; iNOS, inducible nitric oxide synthase; CD80, cluster of differentiation 80; Arg1, Arginase 1.

The effects of VER on LPS‐induced inflammation in mice was further investigated. Lung inflammation was assessed by quantifying neutrophil infiltration using the myeloperoxidase (MPO) activity assay and immunofluorescence of lung sections stained with anti‐MPO antibody. In the LPS‐treated group, the proportion of neutrophils increased significantly. However, the proportion of neutrophils decreased in the VER‐ or DXM‐treated groups (Figures 2A and B). Our single‐cell RNA sequencing results also confirmed that VER inhibited the proportion of neutrophils in ALI mice, however, further studies are needed to evaluate whether VER directly act on neutrophils in ALI mice (Figure S4). In addition, ALI‐induced activation of MPO was significantly inhibited by VER treatment, as evidenced by the decreased MPO expression and activity in the VER‐treated mice. Similar results were observed in the DXM‐treated group (Figure 2C). Several typical proinflammatory cytokines and chemokines in the lung tissues after ALI were also determined. VER and DXM treatments upregulated the IL‐10 levels (Figure 2D). However, the expression of IL‐6 and TNF‐α were significantly decreased in the VER‐ and DXM‐treated groups (Figures 2E and F). In addition, the expression of M1 (CD80 and iNOS) and M2 markers (Arg1 and CD206) in lung tissues were determined using Western blot assay. VER and DXM treatments reduced the expression of the M1 marker but increased that of the M2 marker (Figure 2G) in lung tissues. Moreover, Western blotting revealed that LPS‐induced TNF‐α production was significantly inhibited by VER and DXM (Figure 2G). These results suggest that the inhibition of YAP1 activity alleviates the proinflammatory response and reconstitutes the inflammatory microenvironment.

### 2.5 Inhibition of YAP1 activity promoted M2 polarization in bone marrow‐derived macrophages

**FIGURE 3:**
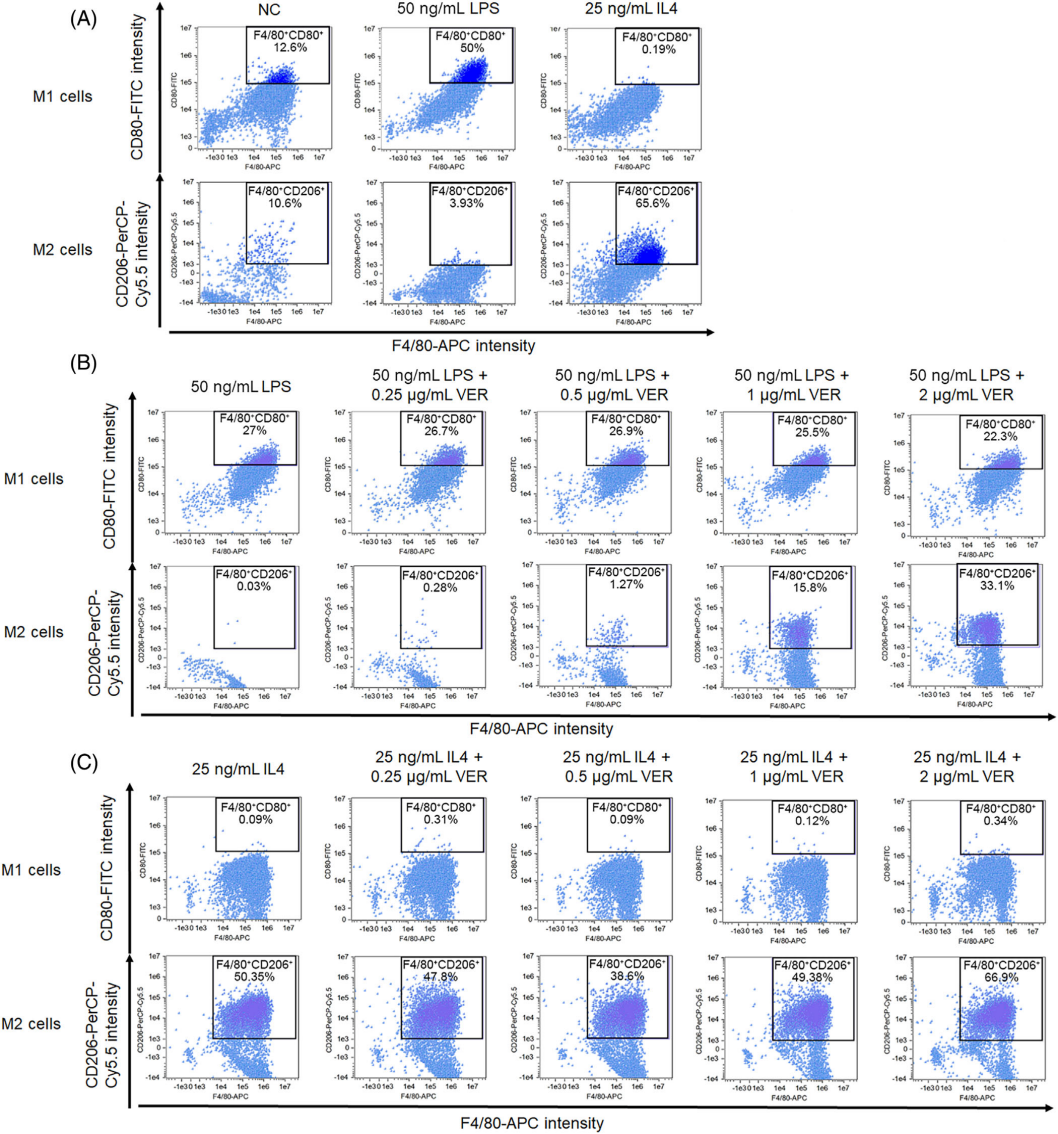
Inhibition of YAP1 activity increased the percentage of M2 macrophages in LPS‐ and IL4‐induced BMMs by flow cytometry assay. (A) Cells were induced by LPS and IL4 for 24 h, respectively. (B) Cells were induced by 50 ng/mL LPS for 24 h, and then treated with 0, 0.25, 0.5, 1, and 2 μg/mL VER for 72 h, respectively. (C) Cells were induced by 25 ng/mL IL4 for 24 h, and then treated with 0, 0.25, 0.5, 1, and 2 μg/mL VER for 72 h, respectively.

In vivo results indicated that the inhibition of YAP1 activity altered the ratio of macrophage subsets in ALI mice. To further explore the role of YAP1 inhibition in macrophage polarization, bone marrow‐derived macrophages (BMMs) were prepared and treated with VER. Flow cytometric analysis showed the percentage of M2 macrophages in total macrophages increased in LPS‐ or IL‐4‐induced macrophages after treatment with VER (Figures 3A–C and Figures S2A–C).

**FIGURE 4:**
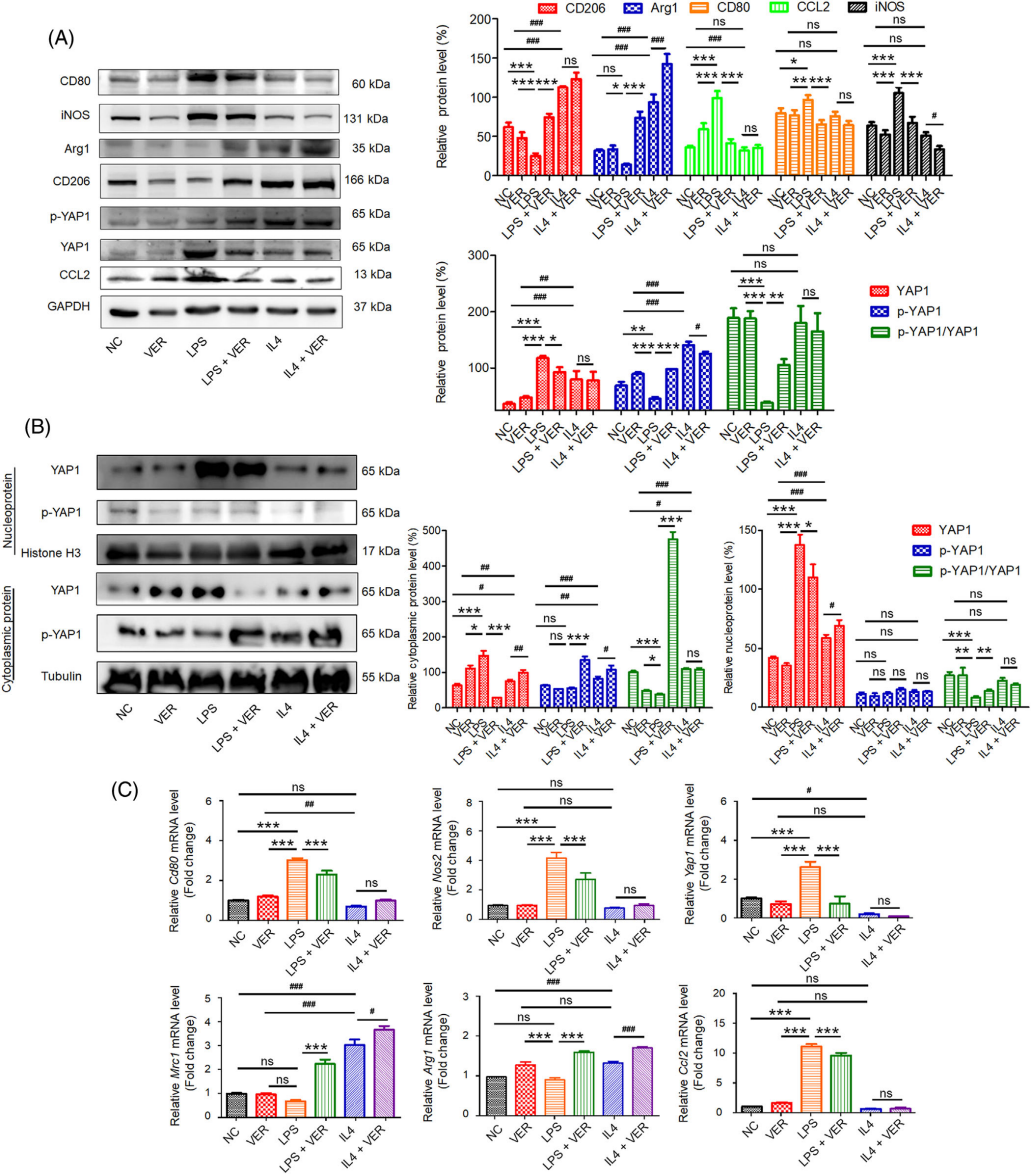
Inhibition of YAP1 activity promoted M2 polarization in LPS‐ and IL4‐induced BMMs. Cells were induced by 50 ng/mL LPS or 25 ng/mL IL4 for 24 h, and then treated with or without 2 μg/mL VER for 72 h. (A) Western blotting and densitometric quantification of CD80, iNOS, CD206, Arg1, p‐YAP1, YAP1, and CCL2. (B) Western blotting and densitometric quantification of p‐YAP1, YAP1 in the nucleus and cytoplasm. (C) The mRNA expression of *Cd80*, *Nos2*, *Mrc1*, *Arg1*, *Yap1*, and *Ccl2* determined by qRT‐PCR. All data are presented as the mean ± SD (*n* = 5). ns, no significance; *p < 0.05, **p < 0.01, ***p < 0.001 compared with LPS‐treated group; ^#^ *p* < 0.05, ^##^ *p* < 0.01, ^###^ *p* < 0.001 compared with IL4‐treated group. CD206, gene symbol: *Mrc1*; iNOS, gene symbol: *Nos2*; CD80, gene symbol: *Cd80*; Arg1, gene symbol: *Arg1*; YAP1, Yes‐associated protein 1 (gene symbol: *Yap1*); CCL2, chemokine (C‐C motif) ligand 2 (gene symbol: *Ccl2*).

Additionally, western blotting demonstrated that VER downregulated LPS‐induced expression of M1 markers (CD80 and iNOS). Compared with the IL4 alone group, the expression of M2 markers (Arg1 and CD206) was significantly increased in IL4‐induced macrophages after treatment with VER (Figure 4A).

Yes‐associated protein (YAP), an effector of the Hippo signaling pathway, localizes to both the cytoplasm and nucleus and influences cell proliferation, stem cell status, and tissue homeostasis.^17^, ^18^, ^19^ We further evaluated changes in YAP localization using Western blotting. No significant changes in p‐YAP1 expression were observed in the nucleus (Figure 4B). In LPS‐induced cells, YAP expression was significantly upregulated in the cytoplasm (Figure 4B). After VER treatment, the YAP expression was inhibited and the level of phosphorylated YAP1 was significantly increased, suggesting that YAP1 activity was inhibited in LPS‐induced BMMs (Figure 4B).

The mRNA expression levels of M1 and M2 markers were also determined. As shown in Figure 4C, VER significantly decreased the mRNA expression of M1 markers in LPS‐induced macrophages; however, it upregulated the expression of M2 markers in IL4‐induced macrophages. An increasing number of studies have reported that C‐C Motif Chemokine 2 (CCL2) plays a critical role in the development of inflammation.^25^, ^26^ In LPS‐induced inflammatory conditions, the levels of CCL2 were significantly elevated. In addition, VER decreased LPS‐induced CCL2 mRNA and protein expression in BMMs (Figures 4A and C). However, no significant changes were observed in IL4‐induced BMMs. Our results suggest that the inhibition of YAP1 activity by VER promotes M2 polarization and downregulates YAP1 expression and activity in BMMs.

### 2.6 LATS1/YAP1 regulated the polarization state of M1/M2 macrophage in BMMs

**FIGURE 5:**
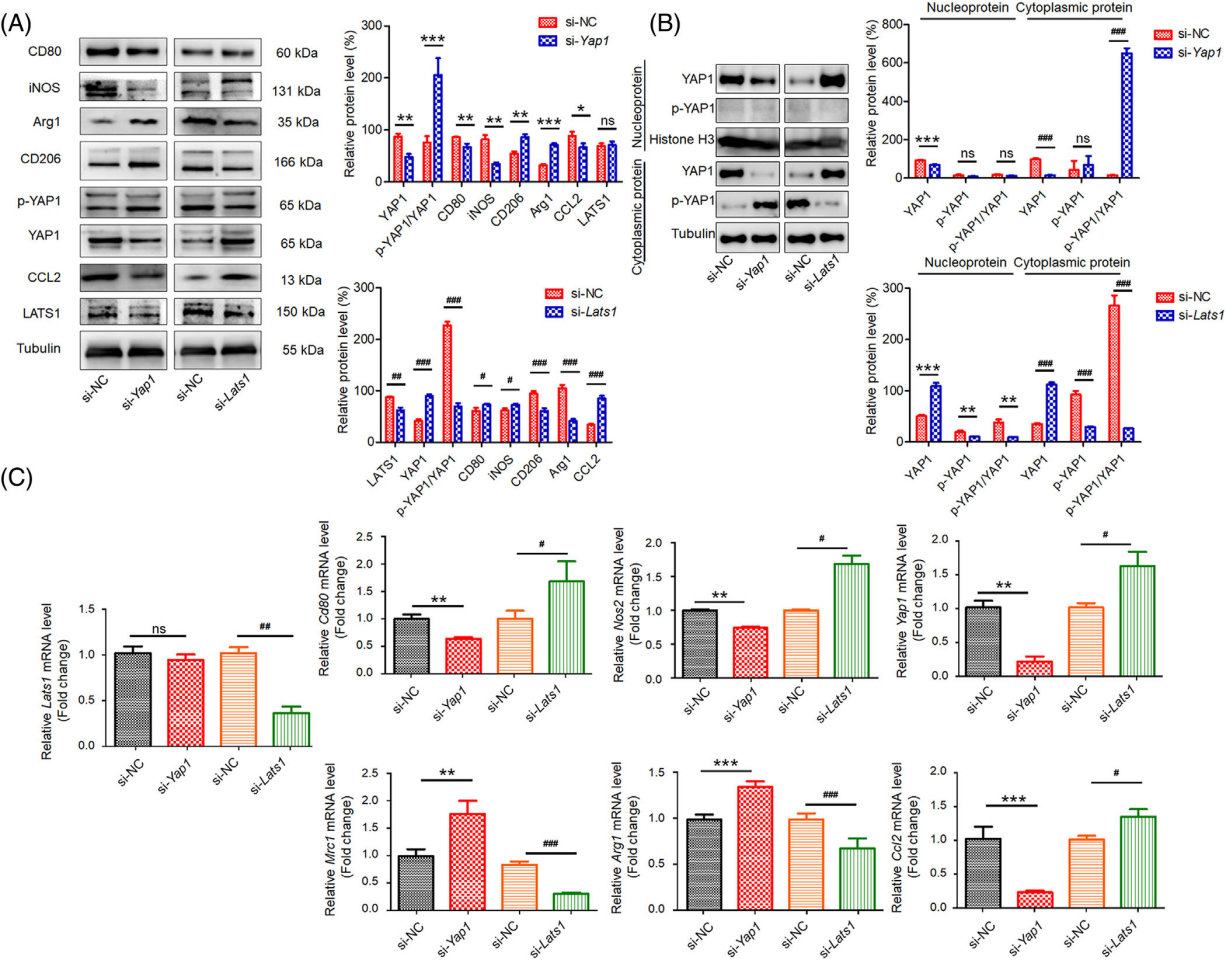
LATS1/YAP1 regulated the polarization state of M1/M2 macrophage in BMMs. Cells were transfected with siRNA‐YAP1 (si‐*Yap1*), siRNA‐LATS1 (si‐*Lats1*), or siRNA negative control (si‐NC). 72 h after siRNA transfection, the cells were collected for qRT‐PCR and western blotting assays. (A) Western blotting and densitometric quantification of p‐YAP1, YAP1, LATS1, CCL2, CD80, iNOS, CD206, and Arg1. (B) Western blotting and densitometric quantification of p‐YAP1, YAP1 in the nucleus and cytoplasm. (C) The mRNA expression of *Yap1*, *Ccl2*, *Cd80*, *Nos2*, *Mrc1*, and *Arg1* determined by qRT‐PCR. All data are presented as the mean ± SD (*n* = 5). ns, no significance; *p < 0.05, **p < 0.01, ***p < 0.001 between two groups. LATS1, large tumor suppressor 1 (gene symbol: *Lats1*).

We further investigated the role of LATS1/YAP1 in the polarization state of M1/M2 macrophage. MST1/2 and LATS1/2 are upstream components of the Hippo signaling pathway in mammals. Direct activation of LATS1 by MST inactivates YAP1.^27^, ^28^ Targeting *Yap1* and *Lats1* mRNA by si‐*Yap1* and si‐*Lats1* siRNA were verified by Western blot, which showed that they significantly abrogated the expression of YAP1 and LATS1, respectively (Figure 5A). When *Lats1* was silenced, YAP1 was dephosphorylated and activated. Thus, YAP1 activity can be inhibited or induced by si‐*Yap1* or si‐*Lats1*, respectively.

To analyze the changes of YAP and p‐YAP in the nucleus and cytoplasm, the nuclear and cytoplasmic proteins were extracted, respectively. When cells were transfected with si‐*Yap1*, YAP1 levels decreased in the nucleus and cytoplasm, however, the expression of YAP1 in the cytoplasm was more significantly reduced. Interestingly, p‐YAP1 levels significantly increased in the cytoplasm, indicating that YAP1 activity was inhibited by cytoplasmic retention after knockdown of *Yap1* (Figure 5B). Furthermore, after silencing *Lats1*, YAP1 levels were elevated in the nucleus and cytoplasm. The levels of phosphorylated YAP1 were inhibited in the cytoplasm, suggesting that YAP1 was activated (Figure 5B).

The mRNA and protein levels of CCL2 were blocked in YAP1 deficiency cells and upregulated in LATS1 deficiency cells, which further confirming that blocking CCL2 expression may be a potential therapy for inhibiting inflammation (Figures 5A and C). Compared with the negative siRNA control group, YAP1 deficiency significantly enhanced the mRNA and protein levels of M2 markers, Arg1 and CD206 (Figures 5A and C). YAP1 deficiency promoted M2 macrophage polarization in BMMs. However, LATS1 deficiency upregulated the mRNA and protein levels of M1 markers, CD80 and iNOS (Figures 5A and C). LATS1 deficiency promoted M1 macrophage polarization in BMMs. Our results suggest that LATS1/YAP1 regulates the polarization of M1/M2 macrophage in BMMs.

### 2.7 Single‐cell RNA sequencing analyzes the subtypes of inflammatory macrophages

We performed single‐cell RNA sequencing to investigate the subtypes of inflammatory macrophages. All CD45^+^ pulmonary immune cells were isolated from the lung tissues of C57BL/6 mice treated with 2.5 mg/kg LPS via endotracheal intubation. A total of 26,790 cells with high quality were analyzed for subsequent cell clustering and annotation after strict quality control. Moreover, the uniform manifold approximation and projection (UMAP) was used to classify each cluster. Totally, 18 distinct clusters were visualized using UMAP algorithm. The top 10 differentially expressed genes (DEGs) from each cluster were unique or shared compared with other clusters (Figure S3A). The cell number was counted in each cluster (Figure S3B). Four major populations in lung parenchyma were identified including macrophages, B cells, neutrophils, and T/NK cells (T cells and natural killer [NK] cells) (Figure S3C). Specifically, 5 of 18 clusters, namely, clusters 1, 4, 9, 15, and 16, belonged to macrophages because they abundantly expressed cluster‐specific markers Cd68 and Marco (Figure S3D). Strong expression of Ighd, Cd19, Cd79a, Igkc, and Iglc3 was detected in clusters 6 and 12, likely representing B cells (Figure S3D). Clusters 2, 5, 7, 10, and 18, corresponding to neutrophils, were identified by the expression of the classic marker Rsad2 (Figures S3D and S4). Clusters 3, 8, 11, 13, and 14 seemed to be similar to T/NK cells, with T cell markers (Cd3g, Cd3e, Cd3d, Cd7, and Nkg7) and NK cell markers (Il2rb and Klrd1) (Figure S3D).

**FIGURE 6:**
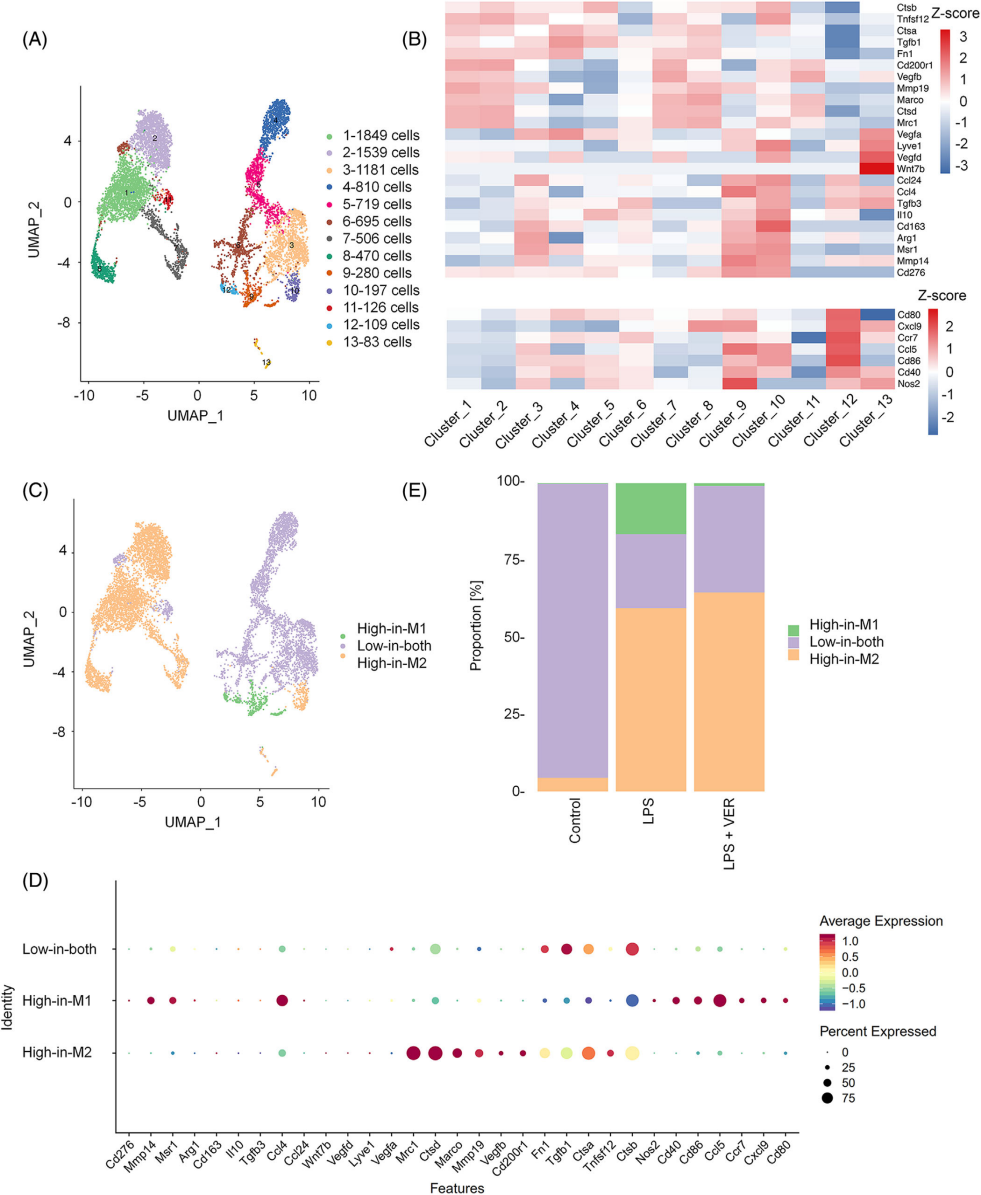
Identification of macrophages by scRNA‐seq. (A) 13 clusters across 8564 cells from lung macrophage subsets on UMAP. (B) Heatmap for the relative expression of M1 and M2 marker genes in each cluster. (C) UMAP exhibiting lung macrophage subtypes in pulmonary tissues. (D) The bubble plot shows marker genes across lung macrophage cell subsets. (E) Relative proportion of cells in control, LPS, and LPS + VER groups.

For macrophages, 13 distinct clusters were observed by UMAP algorithm (Figure 6A). We investigated the expression of M1 and M2 markers in all clusters and found that clusters 1, 2, 7, 8, 10, and 13 revealed high expression of M2 genes, while clusters 9 and 12 expressed high levels of M1 genes (Figure 6B). Notably, clusters 3, 4, 5, 6, 9, and 11 exhibited relatively low expression of both M1 and M2 genes (Figure 6B). This approach led to the differentiation of three cell subtypes, including high expression of M1 genes (High‐in‐M1), high expression of M2 genes (High‐in‐M2) and low expression of M1 and M2 genes (Low‐in‐both) (Figure 6C). Bubble heatmap showed the expression of M1 and M2 marker genes in High‐in‐M1, High‐in‐M2 and Low‐in‐both groups (Figure 6D). During homeostasis, more than 95% of cells exhibited low expression of M1 and M2 genes, while nearly all remaining cells expressed M2 genes in the control group. After LPS administration, the cells expressing M1 genes (16.6%) appeared in the LPS group. The proportion of cells expressing M2 genes was increased by approximately 55% during peak inflammation. This proportion was the highest in the LPS + VER group (64.5%). Moreover, after VER treatment in LPS‐induced mice, the proportion of cells expressing M1 genes was reduced by 15.7% (Figures 6E and S5). Our single‐cell RNA sequencing results further confirmed that inhibition of YAP1 activity influenced macrophage activation and the ratio of the M1/M2 phenotype in lung macrophages. VER treatment reduced the proportion of M1 macrophages but increased the proportion of M2 macrophages in the lungs. These results indicate that inhibition of YAP1 activity by VER restored the balance of macrophage subsets in ALI mice and that M2 macrophages contribute to the repair of lung injury in VER therapy.

We conducted further analysis of the expression of AM and IM markers in 13 macrophage clusters from single cell analysis and found that clusters 1, 2, 7, 8, and 11 showed high expression of AM genes (C1qa, C1qb, C1qc, Ear2, Ear1, Csf2rb, Itgax), while clusters 3, 4, 5, 6, 9, 10, 12, and 13 expressed high levels of IM genes (Cyp11a1, Fcgr1, Cd74, Cx3cr1, Lyz2, Mafb, Cd14, Ly6c1) (Figure S6). Therefore, we observed distinct AM and IM cell subtypes. In ALI mice, the proportion of AMs increased, and the percentage of IMs decreased compared with untreated mice. After VER administration in ALI mice, we observed only a slight increase in AMs and an insignificant reduction in IMs (Figure S7).

**FIGURE 7:**
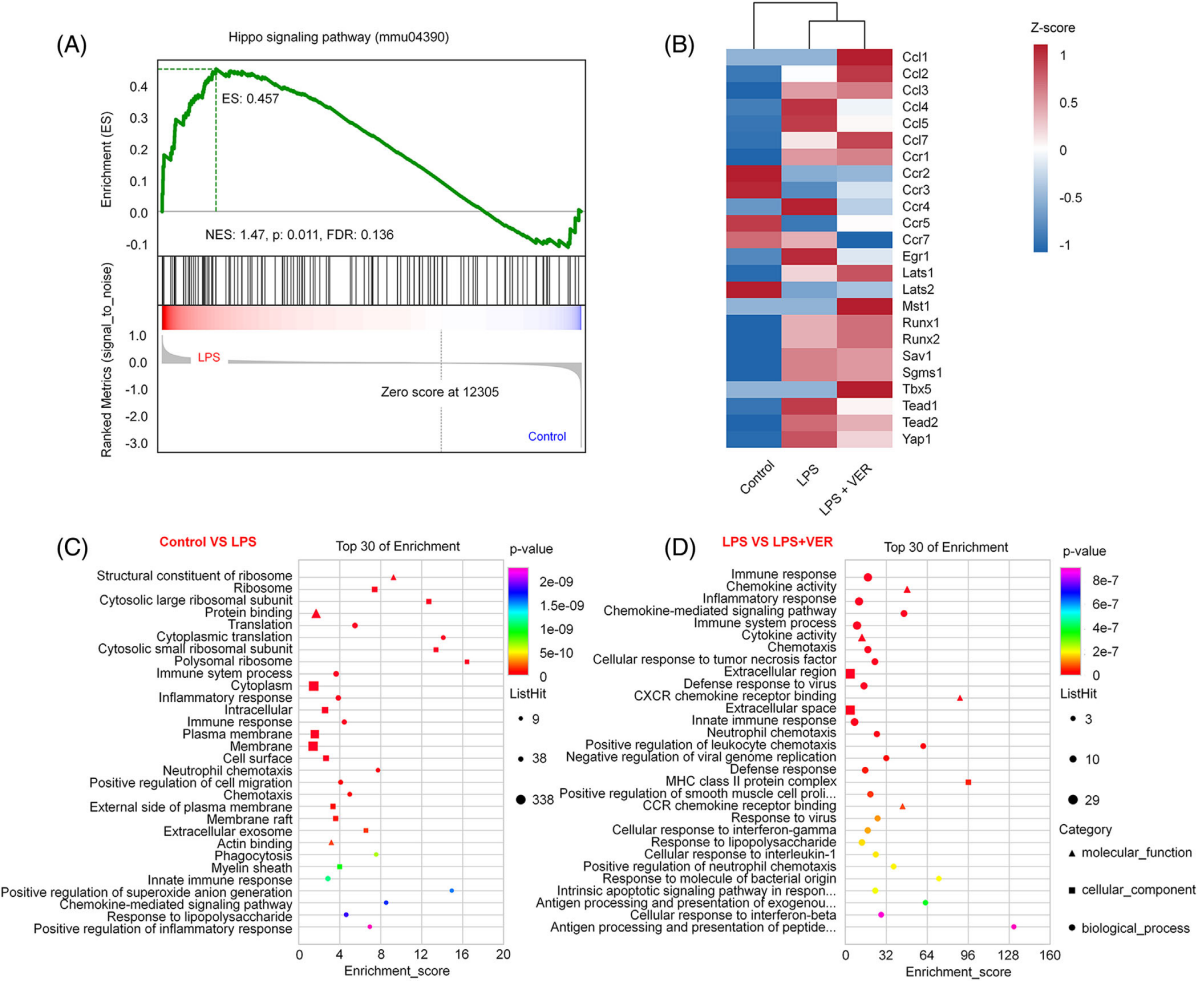
Function and pathway enrichment analysis of differentially regulated genes in LPS‐induced ALI. (A) Gene set enrichment analysis and a signature of the up‐ or downregulated genes between control and LPS groups. NES, normalized enrichment score; FDR, false discovery rate. (B) Heatmap (*z*‐scores rescaled to −1 to 1 as rainbow scale) showing the differentially expressed genes (fold change >1.5 and *p* value <0.05) in the Hippo signaling pathway. (C) KEGG pathway analysis showing differentially expressed genes that were identified between the control and LPS groups. (D) KEGG pathway analysis showing differentially expressed genes that were identified between the LPS and LPS + VER groups (fold change >1.5 and *p* value <0.05).

The GSEA of GO biological processes demonstrated that Hippo signaling pathway (mmu04390) was involved in LPS‐induced ALI (Figure 7A). Then, the heatmap revealed the expression levels of hippo‐pathway‐related genes, which were consistent with our experimental results (Figure 7B). The DEGs were compared with explore the biological function, using bioinformatics analysis by GO and KEGG. For example, KEGG pathway analysis indicated that the DEGs between control and LPS treatment groups were enriched in some important pathways, such as “immune system process,” “immune response,” and “inflammatory response” in the biological process (Figure 7C). After treatment with VER, more immune system related pathways were significantly activated including “inflammatory response,” “immune response,” “chemotaxis,” “chemokine‐mediated signaling pathway,” “neutrophil chemotaxis,” “innate immune response,” “defense response,” and “positive regulation of leukocyte chemotaxis” between LPS and LPS + VER groups (Figure 7D). The above results revealed that VER can activate the immune‐inflammatory response system, promote the potential of M2 macrophages and alleviate LPS‐induced ALI.

## 3 DISCUSSION

The development of ALI includes two phases: the early proinflammatory and the late anti‐inflammatory phase.^10^, ^11^, ^12^ Macrophages consecutively play dual roles in a sequential manner to initiate infection to remove the pathogen as the M1 phenotype, and then restrain inflammation and repair the damaged tissue as the M2 phenotype.^10^, ^11^, ^12^ The balance of M1/M2 macrophage polarization is closely associated with the prognosis of ALI.^10^, ^11^, ^12^

However, various macrophage subtypes exist in pulmonary tissues, and the identification of macrophage subsets is complex and difficult due to the multidimensional, dynamic, and overlapping processes involved in macrophage programing.^29^, ^30^ We applied single‐cell RNA sequencing to identify the polarization state of macrophages from the lung tissues of LPS‐induced ALI in mice. Single‐cell‐level analysis is more accurate than traditional markers of macrophage programming. The M1 markers appeared to be dominant after LPS administration, with a return to the baseline after treatment with VER. The opposite trend was observed on the expression of several M2 activation markers, that had higher expression in the VER‐treated mice compared with control or LPS‐induced model mice. In the LPS‐induced ALI model, VER suppressed M1 macrophage polarization and induce M2 macrophage polarization. Moreover, YAP1 is considered as a transcriptional co‐activator whose activity is determined by the Hippo signaling pathway. We revealed that Hippo signaling pathway (mmu04390) was activated in LPS‐induced ALI. Meanwhile, after administration with VER, the immune‐inflammatory response system was significantly activated. Our results demonstrate that VER promoted M2 macrophage polarization and alleviated LPS‐induced ALI.

Although it has been clarified that YAP1 is a transcriptional coactivator in tumors, the role of YAP1 in macrophages remains to be elucidated.^31^ Accumulating studies demonstrated that YAP1 plays a critical role in regulating M1/M2 macrophage polarization, which controls the pulmonary inflammation process during the development of ALI.^19^, ^20^ We hypothesized that inhibition of YAP1 activity could promote M2 macrophage polarization and alleviate pulmonary inflammation and lung injury following ALI. The main findings of our present study can be generalized as follows: (1) ALI exaggerates pulmonary inflammation and injury accompanied by the downregulated p‐YAP1 and upregulated activity in the lung; (2) inhibition of YAP1 activity attenuates the development of lung injury and improves pulmonary function in ALI mice; (3) inhibition of YAP1 activity suppresses M1 polarization while induces M2 polarization in ALI mice; (4) YAP1 deficiency decreases CCL2 levels and promotes macrophage polarization towards M2 phenotype in BMMs.

VER is a photosensitizer used in photodynamic therapy to treat neovascularization caused by age‐related macular degeneration.^32^ Recent studies have suggested that VER inhibits YAP activation by disrupting its interaction with TEAD, which in turn prevents YAP from promoting oncogenic growth.^23^ VER also disrupts downstream proto‐oncogenes associated with YAP–TEAD, leading to the inhibition of angiogenesis and suppression of growth and migration in human retinoblastoma cells.^33^ Additionally, VER has been found to be effective in inhibiting the expression of YAP and endothelial growth factor receptor, and can enhance the cytotoxic effects of drugs used to kill cells from esophageal and lung cancers.^34^, ^35^ The clinical results of VER photodynamic therapy indicated that it is feasible and safe.^36^ While most reports emphasize the anticancer properties of VER against various tumor types, limited information is available on its association with inflammatory disorders. However, our current study illustrates that VER exhibits robust anti‐inflammatory effects, attenuates lung injury, and improves pulmonary function associated with macrophage polarization. For in vivo experiments, it takes time to spread VER to the lungs. Therefore, VER was injected intraperitoneally into the mice 40 min before LPS administration. For in vitro experiments, IL‐4 (25 ng/mL) or LPS (50 ng/mL) was used to induce M2 or M1 macrophages, respectively. Therefore, we first obtained M1 or M2 macrophages after LPS or IL4 induction, and then used the YAP1 inhibitor VER to confirm the role of YAP1 in M1/M2 macrophage polarization. Overall, our findings suggest that VER is a potential therapeutic option for the treatment of inflammatory conditions.

Interestingly, our results suggested that YAP1 played an important role in regulating the polarization state of pulmonary macrophages during ALI. We found that ALI induced excessive expression of YAP1 and upregulated YAP1 activity in pulmonary macrophages in the lung. In agreement with our studies, silencing *Yap1* or *Lats1* demonstrated the opposite effect, in which YAP1 deficiency improved M2 polarization but LATS1 deficiency induced M1 polarization in BMMs. Overall, these results demonstrate that the role of YAP1 in lung macrophages is involved in the imbalance of M1/M2 polarization and pulmonary inflammation during the development of ALI.

Based on our results, M2 polarization mediated by YAP1 alleviated ALI. However, the exact molecular mechanism by which YAP1 regulates M1/M2 macrophage polarization remains unknown. Several transcription factors, including STAT6, C/EBPB, and IRF4, can upregulate M2‐associated genes, whereas IRF5 is required for M1 polarization and can determine macrophage fate.^37^, ^38^, ^39^, ^40^ These transcription factors may play a role in YAP‐induced macrophage polarization.

According to previous reports, YAP enhances self‐renewal and differentiation of AECIIs into AECIs during lung injury.^41^, ^42^, ^43^ Increased mechanical forces activate YAP in AECIIs cells and promote their proliferation and differentiation into AECIs during alveolar regeneration.^41^, ^42^, ^44^, ^45^ In contrast, previous studies have shown that macrophages can alter YAP expression levels, and in turn, YAP regulates M1 versus M2 macrophage fate in response to local inflammation or gut bacterial infection.^46^ Moreover, our study showed that YAP exacerbates ALI by regulating M1/M2 macrophage polarization in pulmonary macrophages. Thus, YAP plays varying roles in different cell types within the lung microenvironment during ALI development. Targeting YAP in specific cell types is recommended to achieve therapeutic effects against inflammation‐related diseases. Overall, YAP displays diverse functions in multiple cell types, promoting AECII proliferation and differentiation into AECIs, and regulating M2/M1 macrophage polarization. Thus, targeting YAP in specific cell types is recommended for the effective treatment of inflammation‐associated ailments. This “feedback loop” effect of YAP in macrophage fate or function may be a critical “controller” that determines whether the host returns to homeostasis or develops disease. Therefore, we suggest that YAP should be included in the list of key transcription factors and/or co‐activators that manipulate macrophage fate or plasticity.

CCL2, a member of the C‐C chemokine family, is expressed in multiple cell types, such as fibroblasts, endothelial cells, epithelial cells, and monocytes.^25^, ^26^ However, the main source of CCL2 is considered to be from monocytes/macrophages.^25^, ^26^ The migration of monocytes and macrophages to the site of inflammation is partly facilitated by the CCL2/CCR2 chemotaxis gradient. Along with its role in driving monocyte migration, CCL2 signaling also contributes to M2 macrophage polarization.^47^, ^48^ IL‐4 increases CCL2 expression in macrophages, which in turn regulates macrophage polarization extent.^49^ Inhibiting the binding of CCL2 to CCR2 increases the expression of M1‐associated genes, indicating the involvement of CCL2/CCR2 in macrophage polarization.^50^ Our results also suggested that CCL2/CCR2 is closely associated with YAP1 signaling and may be a target for YAP1‐mediated macrophage polarization. However, further research is needed to understand the detailed mechanisms underlying the influence of CCL2 on the polarization of YAP1‐induced M1/M2 macrophages during the development of ALI.

Our studies indicate that inducing YAP1 activity by LPS led to the imbalance of M1/M2 macrophage polarization and elevated levels of proinflammatory cytokines including IL‐10, IL‐6, and TNF‐α. The underlying molecular mechanism by which YAP1 induces the production of proinflammatory cytokines has not been clearly identified. Previous reports show that the binding of YAP1 increases the secretion of IL‐6.^51^, ^52^ In addition, YAP1 promotes the expression of IL‐6 through regulating immune reprogramming in pancreatic cancer.^53^, ^54^ The present study indicates that YAP1 targeted therapy with enhanced IL‐6 expression in macrophages is a promising strategy for treating pulmonary inflammation and subsequent injury during ALI.

In summary, elevated expression of YAP1 in macrophages leads to marked pulmonary inflammation and injury during ALI. Therefore, inhibition of YAP1 activity by VER attenuates lung injury and improves pulmonary function, making it a promising novel therapy for ALI. More studies are needed to reveal the molecular mechanism of YAP1 in regulating M1/M2 macrophage polarization.

## 4 MATERIALS AND METHODS

### 4.1 Chemical and reagent

LPS, VER, and DXM were purchased from MedChem Express (Shanghai, China). Fetal bovine serum was obtained from Inner Mongolia Opcel Biotechnology (Hohhot, China). RPMI 1640, Opti‐MEM medium, and phosphate buffered saline were obtained from Gibco (Grand Island, NY, USA). The IL‐10, IL‐6, and TNF‐α enzyme linked immunosorbent assay kits (ELISA kits) were acquired from Boshen (Yancheng, China) and the MPO detection kit was from BestBio (Shanghai, China).

### 4.2 Induction and treatment of ALI

All the animal experiments were approved by the Institutional Animal Care and Use Committee of Guangzhou Medical University (SYK2016‐0168, GY2021‐135). The C57BL/6 mice were purchased from Guangdong Vital River Laboratory Animal Technology Co., Ltd. The C57BL/6 mice (6–7 weeks old, male, SPF grade) were treated with 2.5 mg/kg LPS by endotracheal intubation to induce an ALI model in vivo. The mice were randomly divided into five groups: namely saline (the control group, only treated with saline); LPS (the model group, only administered with 2.5 mg/kg LPS); VER (only injected with 100 mg/kg VER); LPS + VER (administered with 2.5 mg/kg LPS and treated with 100 mg/kg VER); LPS + DXM (the positive control group, administered with 2.5 mg/kg LPS and treated with 5 mg/kg DXM, a widely prescribed anti‐inflammatory drug). The VER or DXM was injected intraperitoneally into the mice 40 min before LPS administration. Buxco PFT system (DSI, DE, USA) was used for the pulmonary function test in mice 72 h after LPS administration. At the end of the experiment, the mice were anesthetized using sodium pentobarbital and euthanized.

### 4.3 Isolation and identification of macrophages

The BMM was isolated performed according to the protocol as previously described. Bone marrow cells were collected and cultured in RPMI 1640 medium containing 10 ng/mL macrophage colony‐stimulating factor (M‐CSF). After 1 week, the bone marrow cells were differentiated into BMMs. BMMs were identified by the marker of F4/80. Then, the proportion of F4/80 positive cell populations was analyzed by flow cytometry and immunofluorescence staining.

### 4.4 Cell transfection

Lipofectamine 3000 (Invitrogen, MA, USA) were applied for the transfections of si‐*Yap1*, siRNA specific for Yap1 (sense (5′–3′): CGGUUGAAACAACAGGAAUUA; antisense (5′–3′): UAAUUCCUGUUGUUUCAACCG); si‐*Lats1*, siRNA specific for Lats1 (sense (5′–3′): GGUGAAGUCUGUCUAGCAATT; antisense (5′–3′): UUGCUAGACAGACUUCACCTT); or si‐NC (sense (5′–3′): UUCUCCGAACGUGUCACGUTT; antisense (5′–3′): ACGUGACACGUUCGGAGAATT). 72 h after siRNA transfection, the cells were collected for RNA or total protein extraction.

### 4.5 Flow cytometric assay

Isolated pulmonary macrophages were incubated with FITC‐conjugated anti‐mouse CD80 (BD Biosciences, CA, USA), APC‐conjugated anti‐mouse F4/80 (BD Biosciences), and PerCP‐Cy5.5‐conjugated CD206 (BD Biosciences). Analysis was performed by ImageStreamx Mark II flow cytometer (Merck, Darmstadt, Germany). M1 macrophages were classified as F4/80+/CD80+ and M2 macrophages were identified as F4/80+/CD206.

### 4.6 Induction of M1/M2 polarization

Fresh medium containing IL‐4 (25 ng/mL) or LPS (50 ng/mL) was used to induce M2 or M1 macrophages. 24 hours after polarization, M1/M2‐related genes were detected by quantitative real‐time PCR (qRT‐PCR).

### 4.7 Quantitative real‐time PCR assay

The extracted RNA and qRT‐PCR analysis were carried out based on previous methods.^55^ Briefly, total RNA was prepared using an EZ‐press RNA Purification Kit (EZBioscience, CA, USA) and cDNA was synthesized using SimpliAmp Thermal Cycler (Life Technologies, Marsiling, Singapore). qRT‐PCR was performed using a Roche LC 480 II (Roche, Basel, Switzerland). The primer sequences designed for this experiment are listed in Table S1.

### 4.8 Western blotting assay

Western blotting was performed using a universal procedure.^56^ Primary antibodies included anti‐YAP1 (Cell Signaling, MA, USA; 14074T), anti‐phospho‐YAP1‐s127 (anti‐p‐YAP1; ABclonal, Wuhan, China; AP0489), anti‐CCL2 (ABclonal; A7277) anti‐LATS1 (Abcam, CA, USA; ab243656), anti‐CD80 (HUABIO, Hangzhou, China; M1007‐10), anti‐CD206 (HUABIO; ET1702‐04), anti‐Arg1 (HUABIO; ET1605‐8), anti‐iNOS (Abcam; ab178945), anti‐GAPDH (Abcam; ab8245), and anti‐Tubulin (Beijing Ray Antibody Biotech, Beijing, China; RM2003). After HRP‐labeled secondary antibodies (ZSGB‐BIO, Beijing, China; ZB‐2305) were used to bind the primary antibodies, the protein of interest was visualized by the enhanced chemiluminescent substrate SuperSignal (Waltham, MA, USA).

### 4.9 Cytokine assay

The expression levels of IL‐10, IL‐6, and TNF‐α were analyzed with commercial ELISA kits. Briefly, the supernatant of BALF was collected for the ELISA detection. The detailed detection steps were performed according to the manufacturer's protocols.

### 4.10 MPO activity detection

The measurement of the MPO activity was performed using an MPO detection kit. In brief, the lung tissues were isolated and grounded, then the lung homogenates were used for the MPO detection following the manufacturer's protocol.

### 4.11 Histological study

The sections of pulmonary tissues were stained by hematoxylin and eosin according to the standard procedure.^57^ A scoring system was performed to evaluate the pulmonary injuries based on the levels of infiltration of inflammatory cells, disorganization of lung parenchyma, and pulmonary edema. 0, no injury; 1, light injury; 2, intermediate injury; 3, widespread injury; and 4, severe injury. The evaluation of lung injury was performed by two pathologists in a blinded manner.

### 4.12 Wet/Dry ratio and EBA extravasation assessment

At the end of the experiment, the ratio of wet/dry tissue mass was assessed with the left lung's middle lobe. The ratio of wet/dry mass was calculated as wet weights (ww)/ dry weights (dw). For the pulmonary capillary leakage assay, the Evans Blue (EB) were injected into the mice through orbit veins 40 min before collecting the pulmonary tissues. Then, the pulmonary tissues were weighted and soaked in formamide for 72 h. Three days later, the extravasation of EBA was detected by an ultraviolet spectrophotometer.

### 4.13 Biodistribution studies

The C57BL/6 mice (6–7 weeks old, male, SPF grade) were randomly divided into four groups and administered with saline, LPS, VER, and LPS + VER. 72 h after injection, the mice were anesthetized through injection with pentobarbital sodium and euthanized. After that, the hearts, livers, spleens, lungs, and kidneys were collected. The fluorescence intensity of harvested organs was detected by IVIS Lumina III (PerkinElmer, USA) and quantitatively analyzed by Living Image 4.5 software.

### 4.14 Immunofluorescence staining

The slides of pulmonary tissues were incubated with anti‐MPO (1:200; Abcam; ab208670) and anti‐F4/80 (1:200; Abcam; ab6640). Then, slides were reacted with Goat Anti‐Rabbit IgG H&L (FITC) (Abcam; ab6785) or Goat Anti‐Rabbit IgG H&L (Cy3) (Abcam; ab6939) conjugated secondary antibodies. DAPI was used to stain the nuclei. The targeted proteins were observed by confocal microscopy (ZEISS LSM880, Germany).

### 4.15 Single‐cell RNA sequencing

Single CD45+ pulmonary immune cells were isolated by MicroBeads (Miltenyi Biotec, Germany) and performed using the Chromium Controller (10× Genomics, Pleasanton, CA). After single‐cell cDNA libraries were prepared, sequencing was performed using the Illumina platform (Illumina, San Diego, CA). CellRanger version 3.0.0 (10× Genomics) was used for mapping to the Ensembl gene symbols and data quality analysis.

### 4.16 Statistical analysis

All data were expressed as the mean ± standard deviation (SD). Comparisons were analyzed by the Student's *t*‐test. A two‐tailed *p* < 0.05 was considered significantly different.

## AUTHOR CONTRIBUTION

L. L., W. X., and A. S. conceived, designed, and interpreted the study. L. Z., X. Z., N. Z., and J. C. undertook the data acquisition and analysis. X. F., H. C., S. W., and Z. L. were responsible for the comprehensive technical support. L. L., W. X., and C. L. were major contributors in writing the paper. X. Y., F. L., Z. S. C., and A. S. contributed to the inspection of data and final paper. All authors have approved the final version of the manuscript.

## CONFLICT OF INTEREST STATEMENT

Author Zhe‐Sheng Chen is an editorial board member but was not involved in the journal's review or decisions related to this manuscript. The other authors declared no conflict of interest.

## ETHICS STATEMENT

All the animal experiments were approved by the Institutional Animal Care and Use Committee of Guangzhou Medical University (SYK2016‐0168, GY2021‐135).

## Supporting information

Supporting Information

## Data Availability

All data are available from the corresponding authors upon request.
