# Supplementary material for: Inhibition of YAP1 activity ameliorates acute lung injury through promotion of M2 macrophage polarization
Source: MedComm (2020). 2023 Jun 5;4(3):e293. doi: 10.1002/mco2.293 (PMC10242261; doi:10.1002/mco2.293)
Supplement: Supplementary file 1 — Supporting Information [file MCO2-4-e293-s001.docx]

**Supporting information**

**Inhibition of YAP1 activity ameliorates acute lung injury through promotion of M2 macrophage polarization**

Lu Liang^1,#^, Wenyan Xu^1,#^, Ao Shen^1,#^, Xiaomei Fu^1^, Huiyu Cen^1^, Siran Wang^3^, Zhongxiao Lin^1, 4^, Lingmin Zhang^1^, Fangyu Lin^5^, Xin Zhang^4^, Na Zhou^4^, Jishuo Chang^1^, Zhe-Sheng Chen^2,^*, Chuwen Li^1,^*, Xiyong Yu^1,^*

^1^Guangzhou Municipal and Guangdong Provincial Key Laboratory of Molecular Target & Clinical Pharmacology, the State & NMPA Key Laboratory of Respiratory Disease, School of Pharmaceutical Sciences & The Fifth Affiliated Hospital, Guangzhou Medical University, Guangzhou, 511436, PR China

^2^Department of Pharmaceutical Sciences, Institute for Biotechnology, College of Pharmacy and Health Sciences, St. John’s University, Queens, NY 11439, USA

^3^Department of Preventive Dentistry, Affiliated Stomatology Hospital of Guangzhou Medical University, Guangdong Engineering Research Center of Oral Restoration and Reconstruction, Guangzhou Key Laboratory of Basic and Applied Research of Oral Regenerative Medicine, Guangzhou, 510182, PR China

^4^State Key Laboratory of Quality Research in Chinese Medicine, Macau University of Science and Technology, Avenida Wailong, Taipa, Macau, PR China

^5^Department of Ophthalmology, Emory University, B5500 Clinic B, 1365B Clifton Road NE, Atlanta, GA 30322, USA

^#^These authors contributed equally to this work.

*Correspondence to: Xiyong Yu, Chuwen Li and Zhe-Sheng Chen

E-mails: yuxycn@aliyun.com, lichuwen@gzhmu.edu.cn and chenz@stjohns.edu


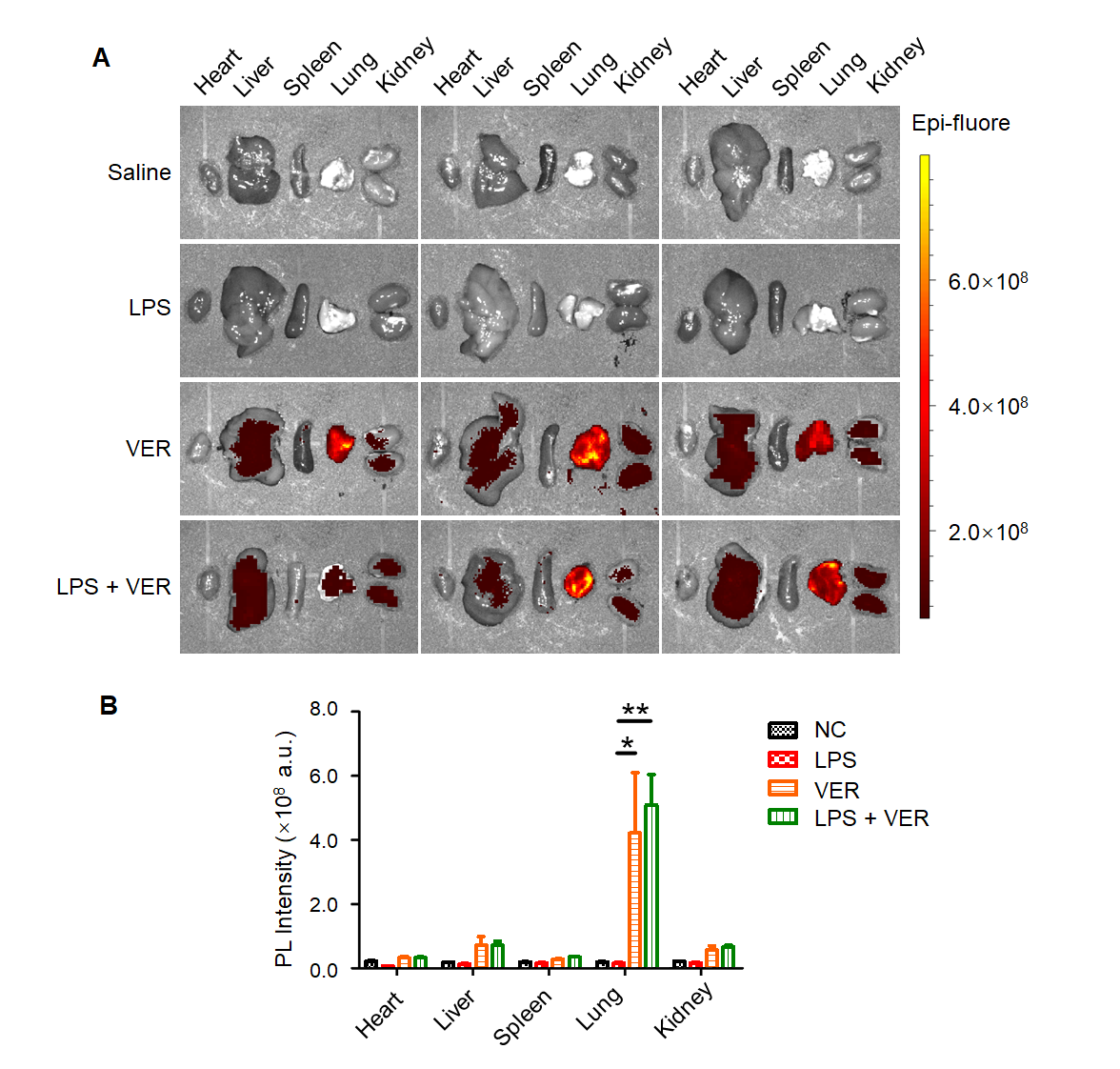


**Figure S1.** The accumulation of verteporfin *in vivo*. Mice were administered with 2.5 mg/kg LPS by endotracheal intubation to induce ALI. Then, 100 mg/kg VER or 5 mg/kg DXM was injected intraperitoneally into the mice 40 min before LPS administration. The major organs were collected at 72 h after LPS administration. (A) Biodistribution of verteporfin in the major organs. (B) The fluorescence quantitative analysis of verteporfin in the major organs. All data are presented as the mean ± SD (n = 5). ns, no significance; **P*< 0.05, ***P* < 0.01, ****P* < 0.001 between two groups.

**
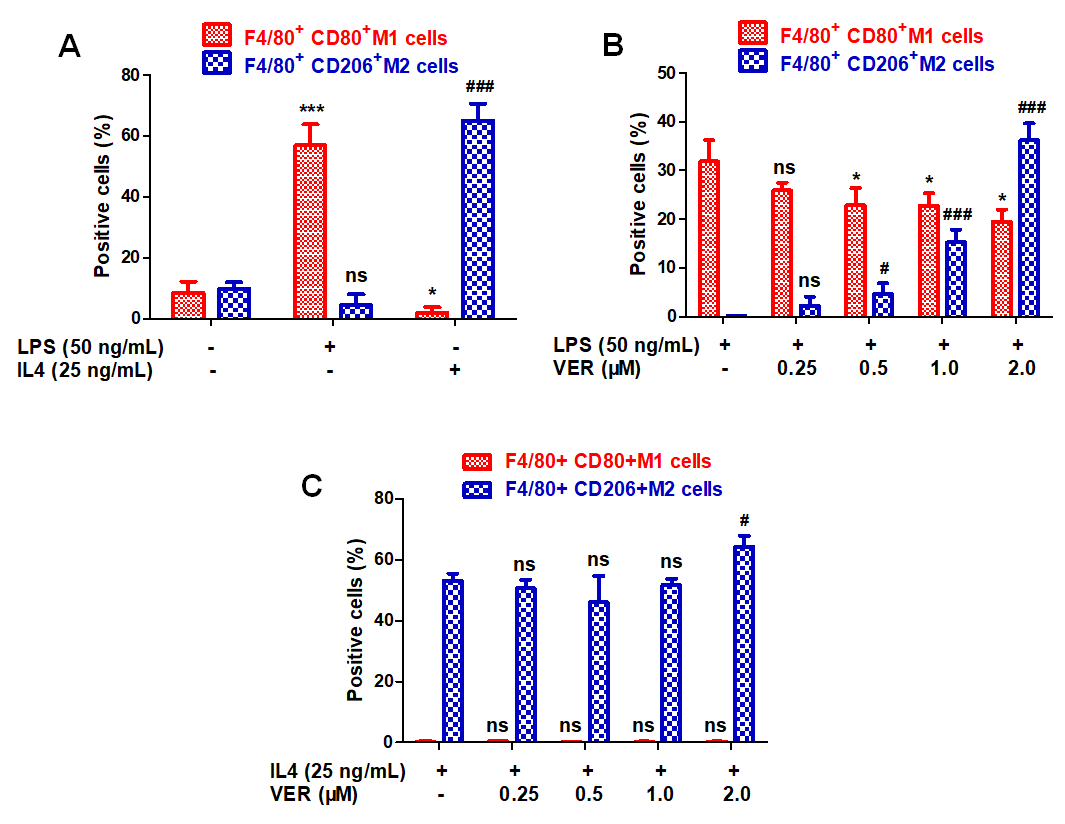
**

**Figure S2.** The percentage of M1 and M2 macrophages were quantitatively analyzed in LPS- and IL4-induced BMM by flow cytometry assay. ns, no significance; **P*< 0.05, ***P* < 0.01, ****P* < 0.001 between two groups in F4/80+ CD80+ M1 cells; ^#^*P*< 0.05, ^##^*P* < 0.01, ^###^*P* < 0.001 between two groups in F4/80+ CD206+ M2 cells.
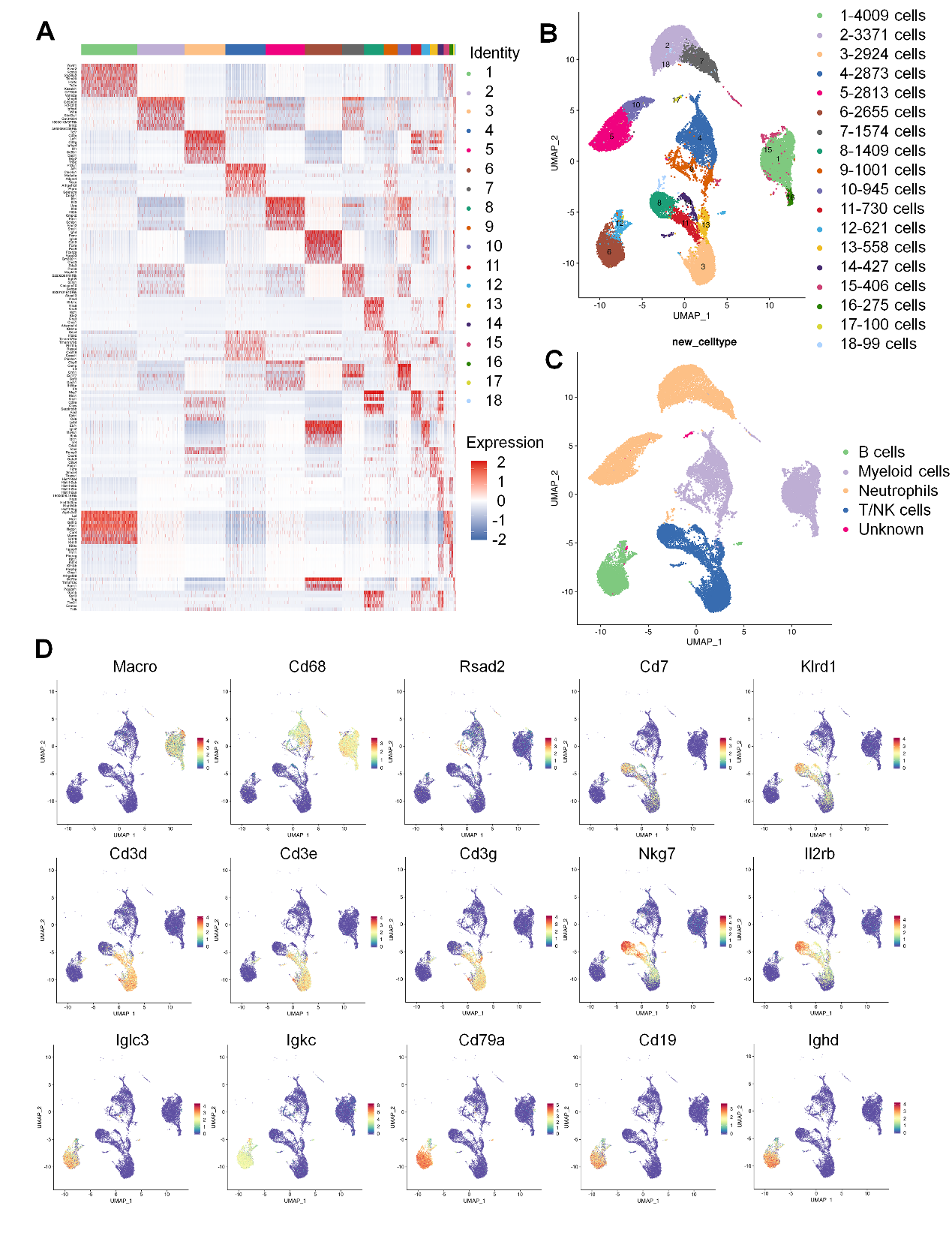


**Figure S3.** Identification of CD45^+^ lung immune cell subsets by scRNA-seq. (A) Scaled expression of the 10 highest differentially expressed genes from each cluster. (B) 18 clusters across 26790 cells from major immune cell population on UMAP. (C) UMAP exhibiting major immune cell subtypes in pulmonary tissues. (D) UMAP showing marker genes across the major immune cell population.

**Figure S4.** UMAP exhibiting immune cell subtypes in pulmonary tissues. Relative proportion of immune cells in control, LPS, and LPS + VER groups.


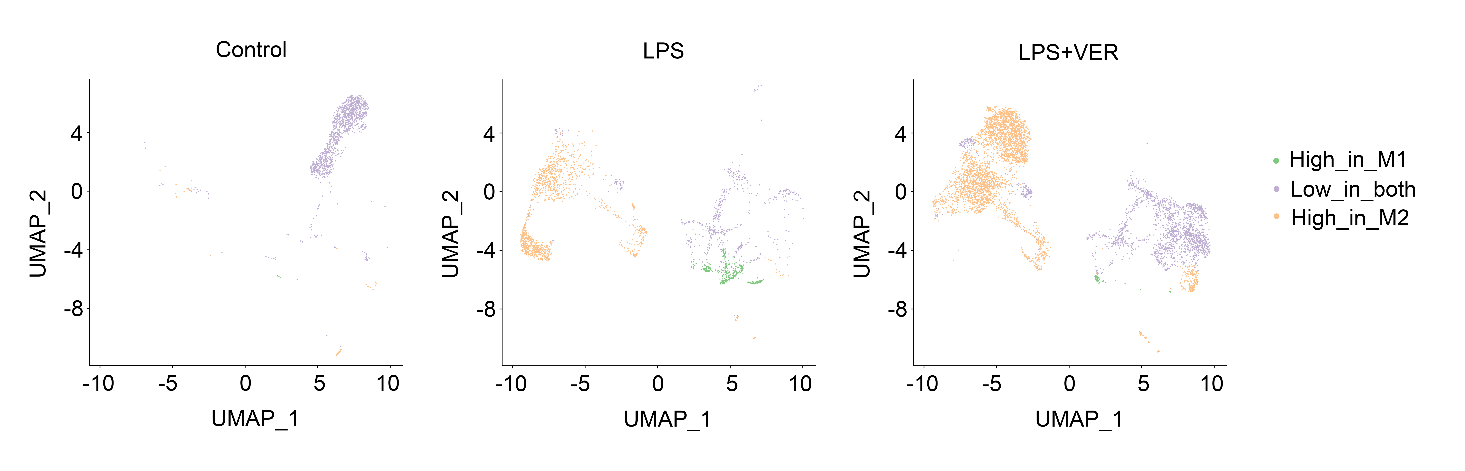


**Figure S5.** UMAP exhibiting lung macrophage subtypes in control, LPS, and LPS + VER groups.

**
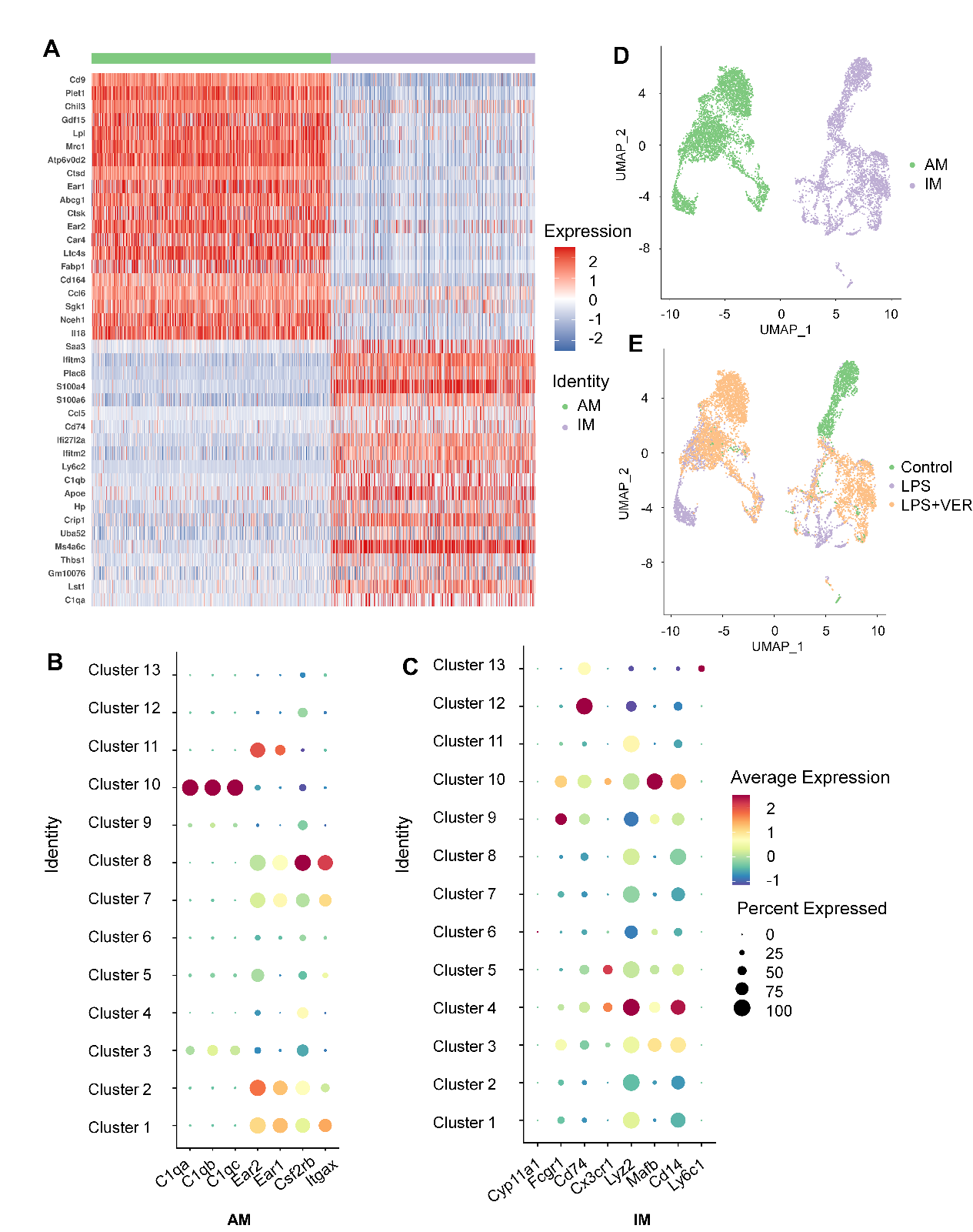
** **Figure S6.** Identification of alveolar macrophage (AM) and interstitial macrophage (IM) subsets by scRNA-seq. (A) Scaled expression of the 20 highest differentially expressed genes from AM and IM cells. (B) The bubble plot shows AM marker genes. (C) The bubble plot shows IM marker genes. (D) UMAP exhibiting major cell subtypes in lung macrophages. (E) UMAP exhibiting the changes of AM and IM cells in control, LPS, and LPS + VER groups.

**
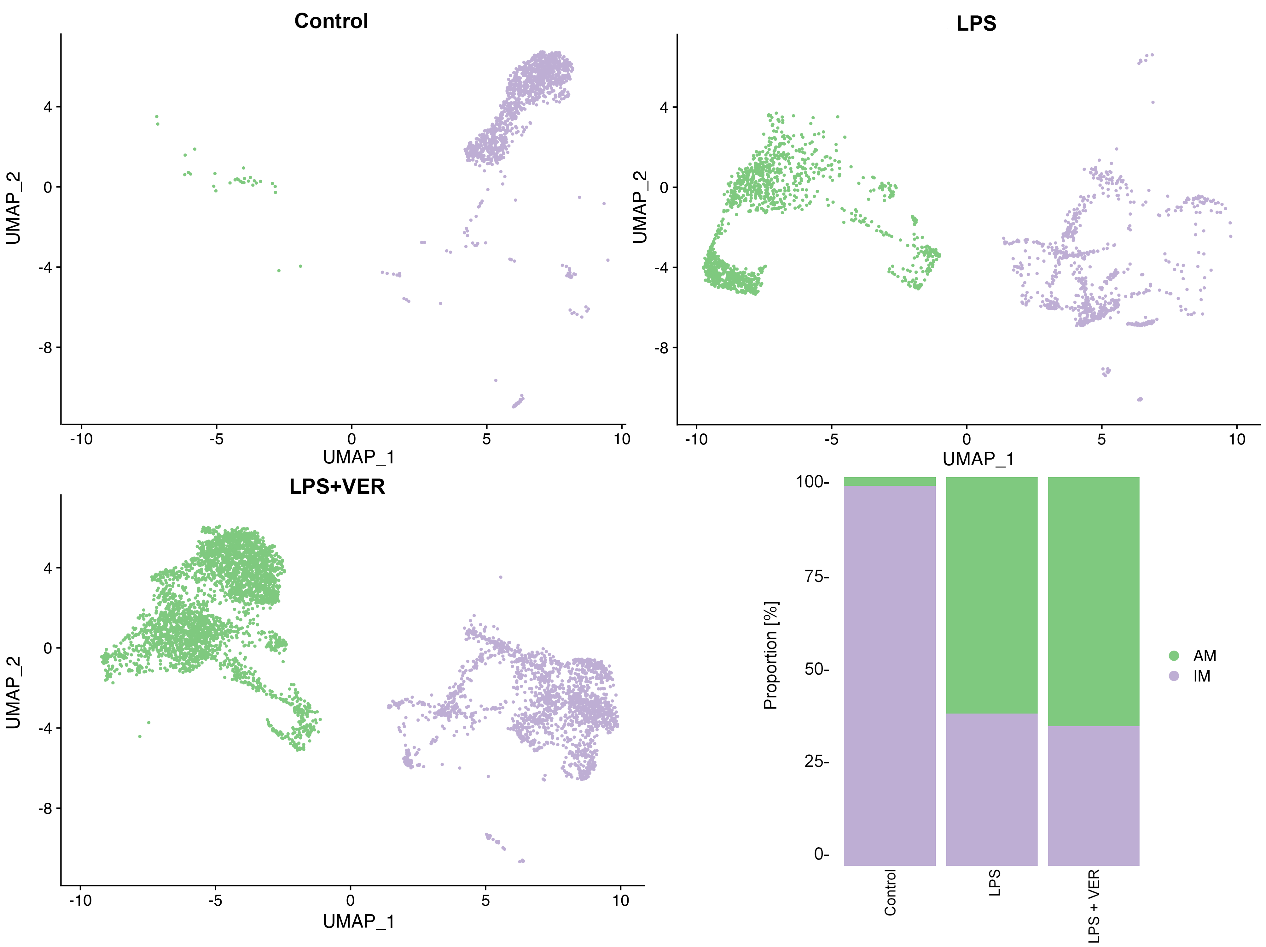
**

**Figure S7.** UMAP exhibiting AM and IM cell subtypes in lung macrophages. Relative proportion of cells in control, LPS, and LPS + VER groups.

**Table S1 Primers for RT-qPCR.**

| Gene | Sequence (5'‑3') |
| --- | --- |
| *Yap1* Forward | TGAGATCCCTGATGATGTACCAC |
| *Yap1* Reverse | TGTTGTTGTCTGATCGTTGTGAT |
| *Ccl2* Forward | TTAAAAACCTGGATCGGAACCAA |
| *Ccl2* Reverse | GCATTAGCTTCAGATTTACGGGT |
| *Cd80* Forward | GCAGGATACACCACTCCTCAA |
| *Cd80* Reverse | AAAGACGAATCAGCAGCACAA |
| *Mrc1* Forward | CTCTGTTCAGCTATTGGACGC |
| *Mrc1* Reverse | CGGAATTTCTGGGATTCAGCTTC |
| *Nos2* Forward | GTTCTCAGCCCAACAATACAAGA |
| *Nos2* Reverse | GTGGACGGGTCGATGTCAC |
| *Lats1* Forward | AAAGCCAGAAGGGTACAGACA |
| *Lats1* Reverse | CCTCAGGGATTCTCGGATCTC |
| *Arg1* Forward | CTCCAAGCCAAAGTCCTTAGAG |
| *Arg1* Reverse | AGGAGCTGTCATTAGGGACATC |
| *Gapdh* Forward | CCCTCAACGACCACTTTGTC |
| *Gapdh* Reverse | AGGGGAGATTCAGTGTGGTG |
